# MULTIMORBIDITY IN LATE LIFE: APPRAISAL, COPING, AND ANTICIPATING CARE NEEDS

**DOI:** 10.1093/geroni/igac059.1125

**Published:** 2022-12-20

**Authors:** Abigail Latimer, Jia-Rong Wu, Patricia McGuire

**Affiliations:** University of Kentucky, Lexington, Kentucky, United States; University of Kentucky, Lexington, Kentucky, United States; University of Kentucky, Lexington, Kentucky, United States

## Abstract

Older adults diagnosed with multiple chronic and serious illnesses are confronted with complex, at times unpredictable, and burdensome medical realities. We present a case study of a 76-year-old woman with heart failure, kidney disease, and arthritis early in her illness course. We use the Theory of Stress and Coping to guide our discussion of her coping appraisals and strategies that promote her psychological and emotional well-being. Although she dismissed her heart failure symptoms as “old age,” she identified pain and disability associated with her arthritis as more of an immediate and serious threat to her autonomy, control, and personal goals. As she is functioning independently and still working, we share her perspective on anticipated caregiving needs and expectations for the last decades of her life. Insights from this case study can inform future research directions and have implications for education and practice.

